# Exploring complex miRNA-mRNA interactions with Bayesian networks by splitting-averaging strategy

**DOI:** 10.1186/1471-2105-10-408

**Published:** 2009-12-10

**Authors:** Bing Liu, Jiuyong Li, Anna Tsykin, Lin Liu, Arti B Gaur, Gregory J Goodall

**Affiliations:** 1 School of Computer and Information Science, University of South Australia, Adelaide, SA 5095, Australia; 2 Centre for Cancer Biology, Hanson Institute, Adelaide, SA 5000, Australia; 3 Norris Cotton Cancer Center, Dartmouth Medical School, Lebanon, New Hampshire 03755, USA; 4 Department of Medicine, The University of Adelaide, Adelaide, SA 5005, Australia

## Abstract

**Background:**

microRNAs (miRNAs) regulate target gene expression by controlling their mRNAs post-transcriptionally. Increasing evidence demonstrates that miRNAs play important roles in various biological processes. However, the functions and precise regulatory mechanisms of most miRNAs remain elusive. Current research suggests that miRNA regulatory modules are complicated, including up-, down-, and mix-regulation for different physiological conditions. Previous computational approaches for discovering miRNA-mRNA interactions focus only on down-regulatory modules. In this work, we present a method to capture complex miRNA-mRNA interactions including all regulatory types between miRNAs and mRNAs.

**Results:**

We present a method to capture complex miRNA-mRNA interactions using Bayesian network structure learning with splitting-averaging strategy. It is designed to explore all possible miRNA-mRNA interactions by integrating miRNA-targeting information, expression profiles of miRNAs and mRNAs, and sample categories. We also present an analysis of data sets for epithelial and mesenchymal transition (EMT). Our results show that the proposed method identified all possible types of miRNA-mRNA interactions from the data. Many interactions are of tremendous biological significance. Some discoveries have been validated by previous research, for example, the miR-200 family negatively regulates *ZEB1 *and *ZEB2 *for EMT. Some are consistent with the literature, such as *LOX *has wide interactions with the miR-200 family members for EMT. Furthermore, many novel interactions are statistically significant and worthy of validation in the near future.

**Conclusions:**

This paper presents a new method to explore the complex miRNA-mRNA interactions for different physiological conditions using Bayesian network structure learning with splitting-averaging strategy. The method makes use of heterogeneous data including miRNA-targeting information, expression profiles of miRNAs and mRNAs, and sample categories. Results on EMT data sets show that the proposed method uncovers many known miRNA targets as well as new potentially promising miRNA-mRNA interactions. These interactions could not be achieved by the normal Bayesian network structure learning.

## Background

MicroRNAs (miRNAs) belong to a group of single-stranded, non-coding RNAs that are 21-23 nucleotides in length [1]. miRNAs target protein coding mRNAs through complementary base-pairing that results in repressing translation and causing mRNA degradation [2,3]. Hundreds of miRNAs have been identified and sequenced in plants, animals, and viruses since the first miRNA, *lin-4*, was discovered in 1993 [4]. As a growing class, it is estimated that miRNAs directly regulate at least 30% of the genes in the human genome [5].

Increasing evidence suggests that miRNAs play important roles in cell differentiation, proliferation, growth, mobility, and apoptosis [6-8]. miRNAs regulate target mRNAs [9], and act as rheostats to make fine-scale adjustments to protein output [10]. Consequently, dysregulation of miRNA function may lead to human diseases, including cancers [11]. However, the functions of most miRNAs and their precise regulatory mechanisms remain elusive. Thus, great efforts have been made to elucidate miRNA functions in recent years.

Extensive studies have proposed the diverse features of miRNA regulation. Mature miRNAs target the 3' untranslated regions (3' UTR) of genes by complementary base-pairing. Furthermore, mature miRNAs can alter the expression of genes by binding to the coding regions as well as the 5' UTR [12,13]. Other regions, known as extended seed and delta seed regions, also contribute to the target selection [14]. miRNAs down-regulating target mRNAs has been widely observed [15,16]. Recent experiments also show that miRNAs up-regulate target mRNAs in some cases [17-20]. In addition, miRNAs may up-regulate target mRNAs in one condition, but repress translation in another condition. For example, *let7 *and the synthetic microRNA *miRcxcr4*-likewise induce translation up-regulation of target mRNAs upon cell-cycle arrest; yet, they repress translation in proliferating cells [17]. The diversity and abundance of miRNA targets result in a large number of possible miRNA regulatory mechanisms. It would be infeasible to test all the possibility with biological experiments in large scale. Alternatively, computational approaches can facilitate experimental validation by producing valid hypotheses from existing data.

Several computational methods have been proposed to study miRNA regulatory mechanisms. Yoon et al. [21] proposed a prediction method for miRNA regulatory modules (MRMs) in which weighted bipartite graphs are adopted to model the binding structures of miRNAs and mRNAs at the sequence level. However, predictions only based on sequence may not be sufficient to determine the complex interactions of miRNA-mRNA pairs. Huang et al. [22] applied Bayesian network parameter learning to infer miRNA-mRNA interactions, while Joung et al. [23] utilized a biclustering approach to discover MRMs. Their methods integrate both sequence information and expression profiles of miRNAs and mRNAs to identify the relevant miRNA-mRNA pairs, thus potentially reduce false discovery rate. Furthermore, Tran et al. [24] applied a rule based method to explore MRMs based on an assumption that miRNAs and mRNAs of a module have similar expression patterns. Similarly, their method uses both sequence information and expression profiles of miRNAs and mRNAs. However, no information of sample categories has been utilized. Considering most biological experiments are designed to compare samples from different phenotypes, conditions, or treatment groups, the sample categories are important for exploring subtle but useful differences. All of the above mentioned methods have not utilized this critical characteristic of comparative design of biological experiments so far. In this study, we will show that without using the information of sample categories, subtle miRNA-mRNA interactions are missed out. In previous work, Liu et al. [25] associated miRNA-mRNA pairs with specific conditions to discover the functional miRNA-mRNA regulatory modules (FMRMs). However, only down-regulation patterns were considered. In this work, we will explore all the possible miRNA-mRNA interactions by taking into account sample categories of comparative designs of biological experiments.

Considering the complexity and diversity of miRNA-mRNA interactions, Bayesian Network (BN) structure learning has the privileges to discover statistically significant miRNA-mRNA interactions from data. It has been used widely for discovering gene regulatory networks [26], but not often for finding miRNA-mRNA interactions yet. In the scenario of BN structure learning, the interactions between miRNAs and mRNAs are defined as dependencies of their states encoded in a graphical representation. In the graph, miRNAs and mRNAs are denoted as nodes and interactions are directed edges. The presence or absence of a directed edge from a miRNA to a mRNA indicates the states of the mRNA are dependent or independent on that of a miRNA. This implies their regulatory relationship. Thus, the dependencies in the graph encode various types of miRNA-mRNA interactions. When the expression data of miRNAs and mRNAs are given, we can use the BN structure learning to capture miRNA-mRNA interactions.

**Figure 1:**
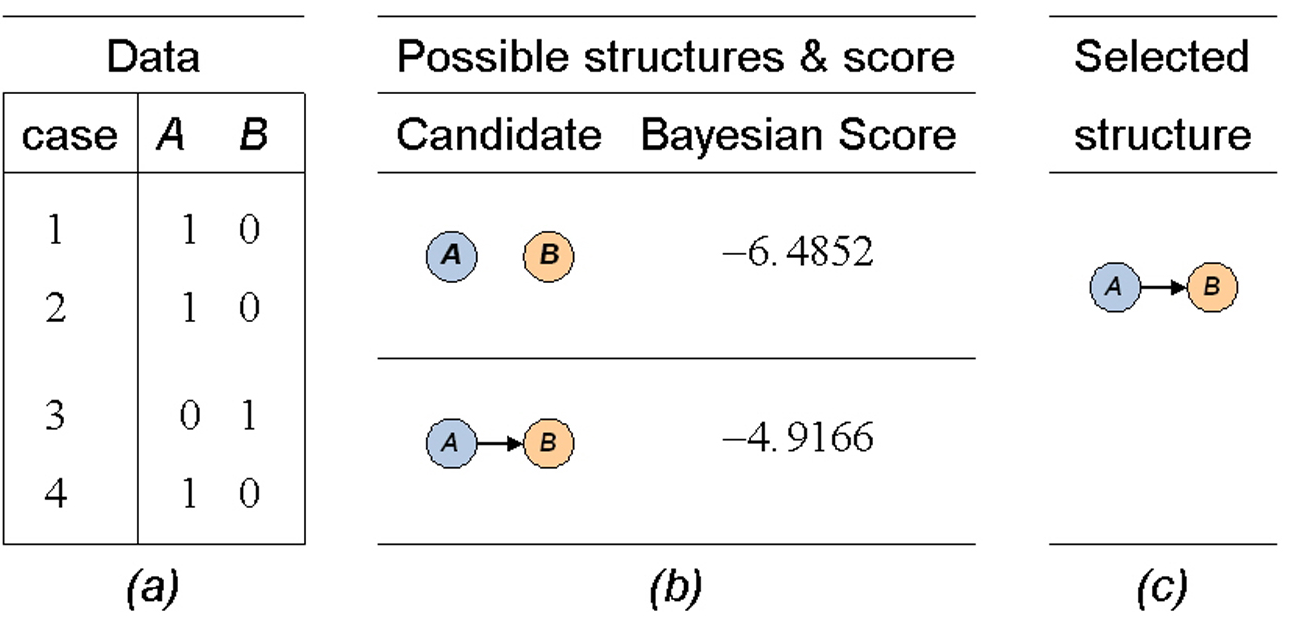
**An example of Bayesian Network structure learning**. (a) Observations of expression of miRNA *A *and mRNA *B *have been discretized to binary values, where 0 denotes under-expressed, and 1 stands for over-expressed. (b) Two possible structures are hypotheses of their relationship. (c) The one that received highest score supported by the data is used to represent the interaction of miRNA *A *and mRNA *B*.

This model-based approach starts by defining the possible structure space, and then followed by a learning procedure to evaluate each structure with a scoring function on the given data [27]. The structure with the maximum score is the one that best depicts the interactions of miRNAs and mRNAs. As a simple example, consider the expression observations of miRNA *A *and mRNA *B *given in Figure 1-(a), where 0 denotes under-expressed and 1 stands for over-expressed. The possible interactions between miRNA *A *and mRNA *B *are no interaction (no edge between *A *and *B*) and miRNA *A *regulates mRNA *B *(a directed edge from *A *to *B*) (Figure 1-(b)). The BN structure learning algorithm calculates a score for each structure against the given expression observations. The one with the highest score, which is best supported by the observations, is chosen to represent the relationship between miRNA *A *and mRNA *B *(Figure 1-(c)). More complex interactions among multiple miRNAs and mRNAs can be factorized to the individual ones according to the probability theory.

**Figure 2:**
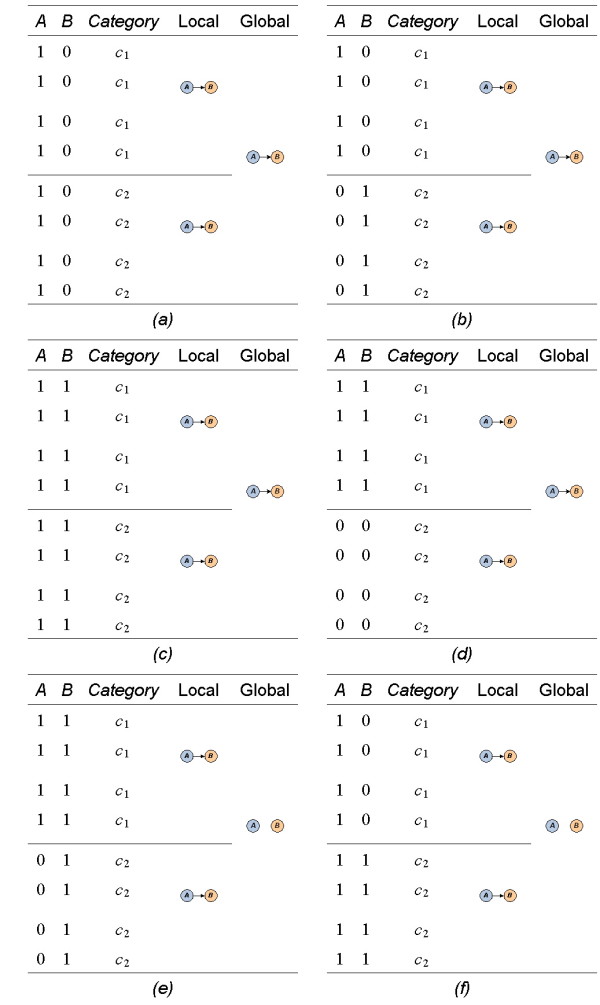
**Various miRNA-mRNA interactions in comparative study**. Three types of observed miRNA-mRNA interactions are: i) miRNAs down-regulate mRNAs (a, b); ii) miRNAs up-regulate mRNAs (c, d); and iii) miRNAs down-regulate mRNAs in one category, but up-regulation appears in another category (e, f). Subtle interactions in (e) and (f) are unrevealing if the analysis ignores the sample categories. Our designed method is able to uncover all types of interactions.

However, under the comparative design of microarray experiments, subtle interactions are unrevealing to a normal BN structure learning method. Demonstrated in Figure 2, six cases represent three observed miRNA-mRNA interactions in different conditions: i) miRNAs down-regulate mRNAs (*a*, *b*); ii) miRNAs up-regulate mRNAs (*c*, *d)*; and iii) miRNAs down-regulate mRNAs in one category, but up-regulation appears in another category (*e*, *f*). The normal BN structure learning is able to capture the interactions showed in cases of (*a*) - (*d*), but not (*e*) and (*f*). In the cases of (*a*) - (*d*), the sample correlation within one category (local correlations), either negative or positive, is consistent with the correlation in the other categories. However, the local correlation is inconsistent with the others in the cases of (*e*) and (*f*). In (*e*), the samples in category *c*_1 _are positively correlated, while negatively correlated in category *c*_2_. Similarly, in (*f*) the samples show negative correlation in category *c*_1_, while positive correlation in category *c*_2_. Without considering the sample categories, the normal BN structure learning fails to capture the interactions, even when there is a strong interaction within a category. Therefore, the subtle interactions among miRNAs and mRNAs remain unrevealing.

In this paper, we present a method to capture complex miRNA-mRNA interactions with BN structure learning for specific conditions. This method discovers the dependency relationship between miRNAs and mRNAs which implies their complex interactions on heterogeneous data sets: miRNA-target binding information, expression profiles of miRNAs and mRNAs. In order to capture all possible interactions, we split expression profiles of miRNAs and mRNAs according to sample categories, and then build Bayesian networks on separate data sets. Interaction networks identified on individual data sets are then integrated by BN averaging procedure. To avoid statistically insignificant results due to small data sets, we employ bootstrapping to achieve reliable inference and integration. We call this strategy splitting and averaging of Bayesian networks (SA-BNs).

To test the SA-BNs approach, we used microRNA and mRNA expression data from the NCI-60 panel of cell lines and focused on miRNA-mRNA interactions potentially involved in the biological process of epithelial to mesenchymal transition (EMT). A number of miRNAs and mRNAs are known to be involved in this process and several miRNA-mRNA interactions have been experimentally verified [28,29]. Compared to the results from a normal BN structure learning, SA-BNs uncover more known miRNA targets as well as promising miRNA-mRNA interactions.

## Methods

**Figure 3:**
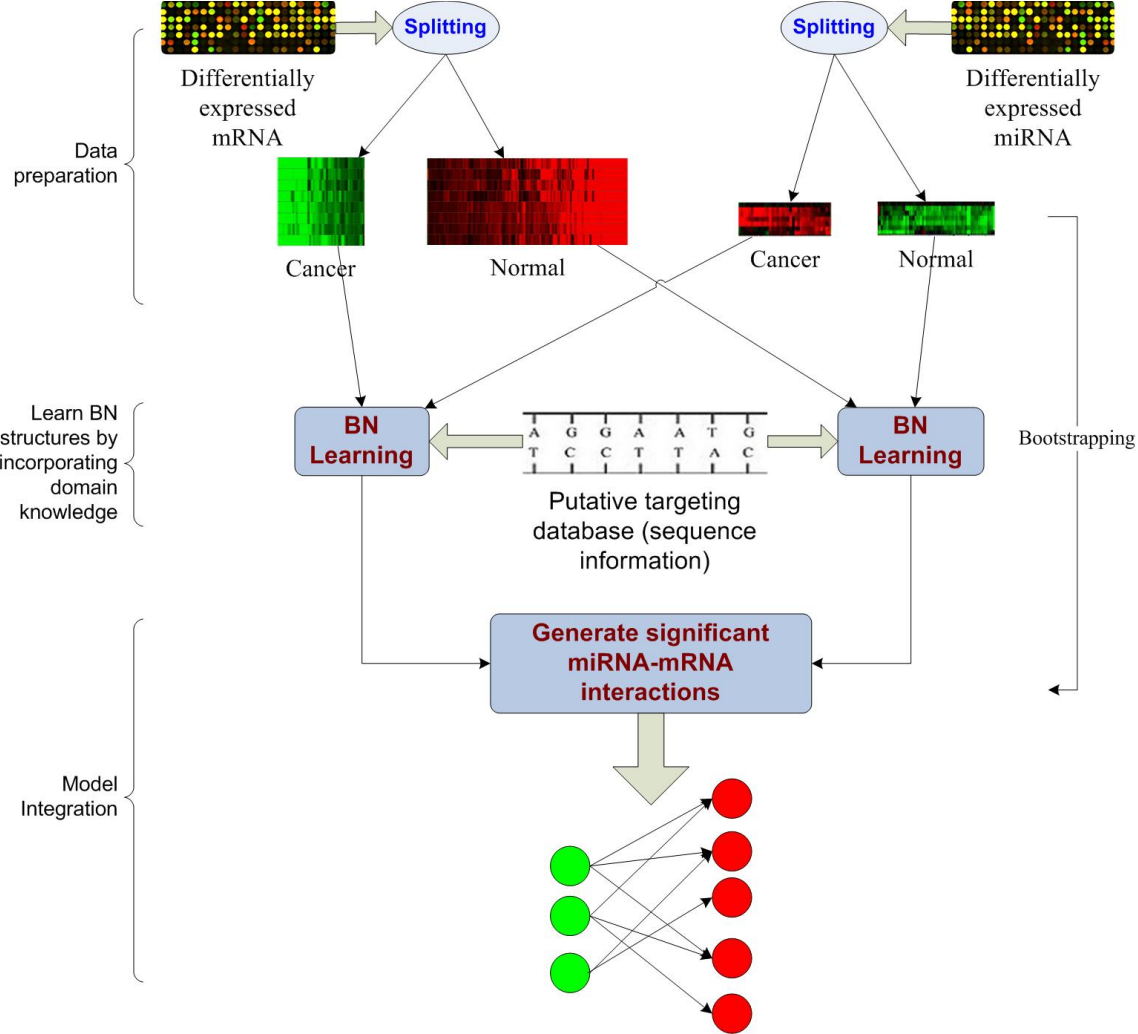
**A flowchart of the design of SA-BNs**. miRNA-target binding information, expression profiles of miRNAs and mRNAs, and sample categories are used to discover the complex miRNA-mRNA interactions.

In this section, we present a model of SA-BNs to discover miRNA-mRNA interactions. The scheme of SA-BNs is shown in Figure 3. After the normalization of expression profiles of miRNAs and mRNAs, differential gene expression analysis identifies a set of miRNAs and mRNAs which are differentially expressed in different conditions under the investigation. Then, we split the expression profiles of identified miRNAs and mRNAs according to categories of samples. Expression profiles of miRNAs and mRNAs have different scales since they are usually profiled with different techniques and platforms. We use discretization as a standardization means for data from different platforms. miRNA and mRNA expression profiles are then integrated for Bayesian network structure learning.

For each sample category, Bayesian network structure learning is used to learn the dependency structures of miRNAs and mRNAs on the discretized profiles. The individual structures learned from data of each category are then integrated into an overall miRNA-mRNA interaction network by the designed BN averaging procedure. In order to control the false discovery, we make use of miRNA-targeting information in the learning process.

We note that the sample size of miRNA or mRNA is usually small in practice. Bootstrapping [30], that is, resampling with replacement, is applied to above procedures for robust inference. The belief confidences of inferred interactions are estimated by a statistic model. This model is to approximate frequency distributions of miRNA-mRNA interactions from bootstrapping. Significant miRNA-mRNA interactions and their confidence scores are thus achieved.

### Annotation

Consider two expression data sets profiling *N *miRNA and *M *mRNA transcripts across *S *samples, respectively. Those samples belong to *C *different categories, either phenotypes, conditions, or treatment groups. Let *i*, *j*, where 1 ≤ *i *≤ *N *and 1 ≤ *j *≤ *M*, be the indices denoting the particular miRNA and mRNA. Let **x **= {*x*_*i*_} and **y **= {*y*_*j *_} be the vectors of miRNAs and mRNAs, and *S*_*k *_be the number of samples of category *k*, where 1 ≤ *k *≤ *C*.

According to the sample categories, we reconstruct the two data sets of miRNA and mRNA to *C *data sets {*D*_*k*_}. Each reconstructed data set *D*_*k *_is composed by *S*_*k *_samples profiling miRNAs **x **and mRNAs **y **for category *k*. That is, *D*_*k *_has *S*_*k *_vectors, and each contains *N *+ *M *variables, {**x**, **y**}, denoting miRNAs and mRNAs. We are interested in interactions between **x **and **y **supported by the experiment data. Assume miRNAs are independent to each other, and so as to mRNAs. The miRNA-mRNA interactions are represented as directed bipartite structures. Thus, we shall explore the relationships between **x **and **y **given data sets {*D*_*k*_} under the constraint of miRNA-targeting information.

### Design of SA-BNs

The above question can be modeled as learning Bayesian network structures of miRNAs and mRNAs under topology constraints given the observed data sets. That is, to identify a graph *G*^*h *^depicting the miRNA-mRNA interactions which are best supported by the given data sets {*D*_*k*_}. We use *h *to denote a hypothesis. A graph *G*^*h *^= {**x**,  **y**,  *E*} encodes the dependencies between vertices **x **and **y **with directed edges *E*, whereas no edges mean independence between vertices. We denote the event of presence of an edge between variables *x*_*i *_and *y*_*j *_with *F*_*ij *_. Our objective is to find the probability *p*(*F*_*ij*_) from the inferred graph *G*^*h *^given data {*D*_*k*_}.

{*D*_*k*_} are mutually exclusive to each other after splitting, we have ∑_*k*_*p*(*D*_*k*_) = 1. According to the total probability theorem, the probability *p*(*F*_*ij*_) is expressed as(1)

It indicates that the inference of an edge between two variables is decomposable by averaging its probabilities deduced from individual data sets. By introducing the graph *G*^*h *^learned from data set *D*_*k*_, Equation (1) can be further extended to(2)

where

By Bayesian theorem, the posterior probability *p*(*G*^*h*^|*D*) is calculated by multiplying the prior probability *p*(*G*^*h*^) with the likelihood *p*(*D|G*^*h*^) as(4)

Substitute Equation (4) into Equation (3), we have(5)

The prior probability *p*(*D*_*k*_) in Equation (3) is eliminated in Equation (5). It indicates that the presence of an edge is independent of the data set conditioned on the graph learned from the data set.

We adopt the BNs averaging procedure for each data set in order to alleviate the overfitting which results from the small sample size of available data. *l *graphs with the highest confidence inferred from the data set are averaged at the local level. We further extend Equation (5) to(6)

Thus, the stable inference of interactions between miRNAs and mRNAs given multiple data sets can be achieved. We summarize the procedure of computing *p*(*F*_*ij*_) in the following algorithm.

#### Algorithm 1: Calculating the interaction belief confidence *p*(*F*_*ij*_) of two sets of variables given data sets

Function: Cal_InteractionBelief()

Input:

*C *- number of data set

*D*_*k *_- data sets

*l *- number of candidate graphs from data set *D*_*k*_

*I *- index of parents (miRNAs)

*J *- index of descendants (mRNAs)

*p *- prior probability of a graph

Output:

*p*(*F*_*ij*_) - the interaction belief set of parent *i *to descendant *j*

Cal_InteractionBelief(*C*, *D*_*k*_, *l*, *I*, *J*, *p*)

{

   for *k *in 1 to *C*

       = graph_search(*D*_*k*_, *I*, *J*);/* Given *D*_*k*_, search for *l *graphs with the maximum likelihood *p*(*D*|*G*^*h*^) within the constrained graph space. The graph space is constrained by miRNA-targeting information coded by index *I *and *J*. Discussed in section Learning BN structures with constraints of domain knowledge.*/

   end

   for *i *in *I*

      for *j *in *J*

         *p*(*F*_*ij*_) = Σ_*k*_*Σ*_*l*_*p*(*Fij*|)*p*(*D*_*k*_|)*p*()

      end

   end

}

In Algorithm 1, we constrain the structure learning with miRNA-targeting information. In the following two sections, we discuss the constraints, then present a statistical model to estimate the confidences of inferred interactions.

### Learning BN structures with constraints of domain knowledge

The learning procedure of BNs is computationally consuming. The exhaustive search for the structure that best fits the observations is feasible only when there are a few genes. The space of possible structures grows hyper-exponentially with the number of genes. It has been shown that learning the global optimal BN is NP-complete [31]. Heuristic algorithms, such as hill climbing, can be used to search the state space efficiently. However, heuristic methods usually find a local optimal solution instead of the global one. This largely limits applications of BNs in real world.

An alternative solution to this problem is to constrain the searching space by integrating domain knowledge. It has been suggested that the utilization of domain knowledge can bias the searching space and lead to near optimal solutions [32]. Some methods have been proposed to explore gene regulatory networks by combining prior domain knowledge [33-36]. To a specific research question, the domain knowledge provides the problem-solving preferable constraints to the state space of the particular problem by knocking out obviously unreasonable states without losing the coverage. It may lead to improved network structures in short time [37].

We are interested in the regulatory relationship between miRNAs and mRNAs. The assumption of miRNAs regulating mRNAs constrains the topologies of miRNA-mRNA interactions to be directed bipartite graphs. This constraint reduces the searching space greatly. Furthermore, miRNA target information based on sequence complementary base-pairing provides another biological constraint to the topology. Many targeting databases can be used to construct the topology, for example, miRBase [38], PicTar [39], and TargetScan [40].

We use the miRNA target information from the target database to constrain the searching space of BNs. The putative target relation of miRNAs and mRNAs deduced from the target database is used as an initial structure of miRNA-mRNA interaction network. In Algorithm 1, this structure is given by variable *I *and *J*. *I *denotes the index of parents (miRNAs), and *J *denotes the index of descendants (mRNAs). Function *graph_search*(*D*_*k*_, *I*, *J*) searches bipartite graphs defined by *I *and *J *for the graphs that have maximum likelihood. Remove operation only is used in this function. It removes the edges one by one in the graph space constrained by *I *and *J*. By this way, we can constrain the searching space within the given putative targeting space. Generally, this space is relatively sparse, and hence the computational complexity is reduced. Therefore, we are able to use an exhaustive searching algorithm to discover the optimal solutions within the given space.

### Generating highly confident interactions by integrating knowledge through bootstrapping

Unstable estimation caused by small number of samples is another challenge to BNs. A typical microarray experiment usually includes a large amount of genes and a small number of samples. The small number of samples rarely support statistically significant discoveries. BNs implement a model averaging procedure to average over several candidate solutions to obtain the optimal one. The confidence is estimated by bootstrapping. Averaging and bootstrapping provide BNs a reliable way to analyze data sets with the small size of samples. In our methods, we innovatively improve the methods for belief estimation. We use bootstrapping in the above procedures to estimate the confidence of discovered interactions. Let *n *be the number of bootstrapping, *q*_*ijk *_be the event of learning the interaction between miRNA *i *and mRNA *j *on the local data set *D*_*k*_. Assuming each learning process *q*_*ijk *_is a stochastic process, we approximate the whole learning process as a Bernoulli experiment where *q*_*ijk *_= 1 when miRNA *i *targets mRNA *j *learned from *D*_*k*_, otherwise *q*_*ijk *_= 0. Thus, *q*_*ijk *_follows a binomial distribution *q*_*ijk*_*~B*(*n*, *p*), where *p *is the probability of *q*_*ijk *_= 1. With a reasonable assumption, *p*(*q*_*ijk *_= 1) = *p*(*q*_*ijk *_= 0) = 0.5 is used in the design.

At the integration stage by averaging, the interactions from local data set *D*_*k *_are aggregated. The interaction of miRNA *i *and mRNA *j *learned through multiple data sets, denoted as *Q*_*ij *_= ∑_*k*_*q*_*ijk*_, also follows a binomial distribution *Q*_*ij*_*~B*(*kn*, *p*). Adopting this statistical model, we are able to extract the learned interactions at significant levels.

## Results

In this section, we provide an analysis of miRNA-mRNA interactions for EMT data with the SA-BNs method.

EMT is part of processes of tissue remodeling during embryonic development, wound healing, and an essential early step in tumor metastasis [41]. Several molecular and cellular functions are involved in turning an epithelial cell into a mesenchymal cell. It requires alterations in morphology, cellular architecture, adhesion, and migration capacity [42]. In this work, we use the proposed computational method to discover miRNA-mRNA interactions for EMT.

### Data sources

Our method integrates heterogeneous data to discover the interactions of miRNAs and mRNAs. These data include miRNA targeting information and expression profiles of miRNAs and mRNAs.

Several databases provide the putative targets of miRNAs [38-40]. We use miRBase [38] in this work because it gives more target predictions compared with experimentally supported databases. It allows our methods to produce relatively more hypotheses in a reasonable range. miRNA expression profiles for the NCI-60 panel of 60 cancer cell lines were from Gaur et al. [43]. They are available at the NCI/DTP database http://dtp.nci.nih.gov/mtweb/search.jsp. The mRNA expression profiles for NCI60 were downloaded from ArrayExpress http://www.ebi.ac.uk/arrayexpress, accession number E-GEOD-5720. Cell lines categorized as epithelial (11 samples) and mesenchymal (36 samples, one is not available) were used for this work.

### Identifying differentially expressed miRNAs and mRNAs

We focus on the differentially expressed miRNAs and mRNAs in epithelial and mesenchymal samples. Applying the Welch *t*-test with 10, 000 times permutation, we identified 8 miRNAs (Table 1) that are differentially expressed at significant levels (*p*-value < 0.05, adjusted by Benjamini & Hochberg (BH) method). Using a similar method, 3556 probes of mRNAs (Additional file 1) are differentially expressed at significant levels (*p*-value < 0.05 without adjustment).

**Table 1:** Differentially expressed miRNAs for EMT

miRNA	Welch *t*-statistics	*p*-value	adjusted *p*-value
miR-200c	-14.0734	1.00 × 10^-4^	1.00 × 10^-4^
miR-141	-11.3564	1.00 × 10^-4^	1.00 × 10^-4^
miR-200b	-9.3313	1.00 × 10^-4^	1.00 × 10^-4^
miR-200a	-7.4501	1.00 × 10^-4^	1.00 × 10^-4^
miR-155	6.7720	2.00 × 10^-4^	4.00 × 10^-4^
miR-140	6.6536	1.00 × 10^-4^	4.00 × 10^-4^
miR-203	-5.7669	1.00 × 10^-4^	0.0031
miR-146	4.7355	7.00 × 10^-4^	0.0229

Applying the Welch *t*-test with 10, 000 times permutation, we identified 8 miRNAs that are differentially expressed at significant levels (*p*-value < 0.05, adjusted by Benjamini & Hochberg (BH) method).

miRBase target V5.0 [38] is used to build the putative target pairs between the differentially expressed miRNAs and mRNAs. 1030 pairs of miRNA-mRNA are linked, comprising 6 miRNAs (miR-200c, miR-141, miR-200b, miR-200a, miR-155, and miR-203) and 610 probes for 460 unique mRNAs.

**Figure 4:**
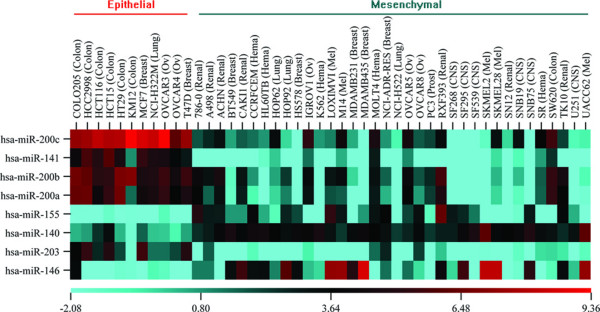
**The heatmap of differentially expressed miRNAs**. The differentially expressed miRNAs are clustered by sample groups and p-values. The identified miRNAs show distinct patterns between epithelial and mesenchymal samples.

Figure 4 is the clustered heat map for the differentially expressed miRNAs across the epithelial and mesenchymal samples. The heat map indicates that the identified miRNAs show distinct patterns between epithelial and mesenchymal samples.

### Discovering and validating miRNA-mRNA interactions with SA-BNs

To integrate miRNA and mRNA data profiled by different platforms, we discretized the data sets to binary values standing for up-regulation and down-regulation. We use the median of each array as the cut-off. The two discretized data sets were merged together sample wise, and then split to two data sets by sample categories, such as epithelia and mesenchymal. It is corresponding to the constant *C *in Algorithm 1. SA-BNs are then used to investigate the miRNA-mRNA interactions on the discretized EMT data sets with 1000 times bootstrapping. Confidences of interactions are estimated accordingly. As a result, we identified 231 statistically significant interactions which comprise 127 unique mRNAs and 6 miRNAs for EMT (Additional file 2).

#### Correlation test suggests both direct and indirect regulations discovered

**Figure 5:**
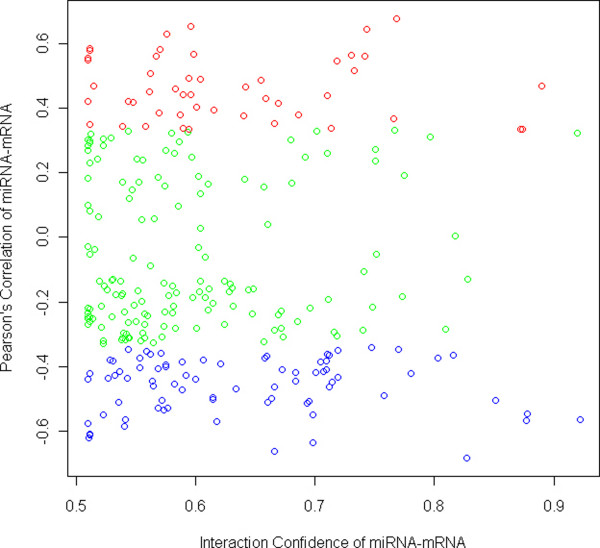
**Pearson's correlation of miRNA-mRNA pairs vs. significant confidences of their interactions discovered by SA-BNs**. The x-axis shows the confidence of interactions, ranging from 0 (the least confident) to 1 (the most confident), while only the statistical significant scale remains (p-value < 0.05). The y-axis shows the sample correlations of identified miRNA-mRNA pairs. The red and blue points are miRNA-mRNA pairs which are correlated at the significant level (p-value < 0.05). It shows that the identified interactions are largely correlated, either negatively or positively, suggesting direct interactions and indirect interactions correspondingly.

The discovered interactions can be categorized to negatively and positively correlated miRNA-mRNA pairs. Figure 5 shows the Pearson's correlation of miRNA-mRNA pairs vs. significant confidences of interaction discovered by SA-BNs. It shows that the discovered miRNA-mRNA pairs are largely correlated, either negatively or positively. The negatively correlated miRNA-mRNA pairs suggest direct interactions, while the positively correlated ones suggest indirect interactions.

**Figure 6:**
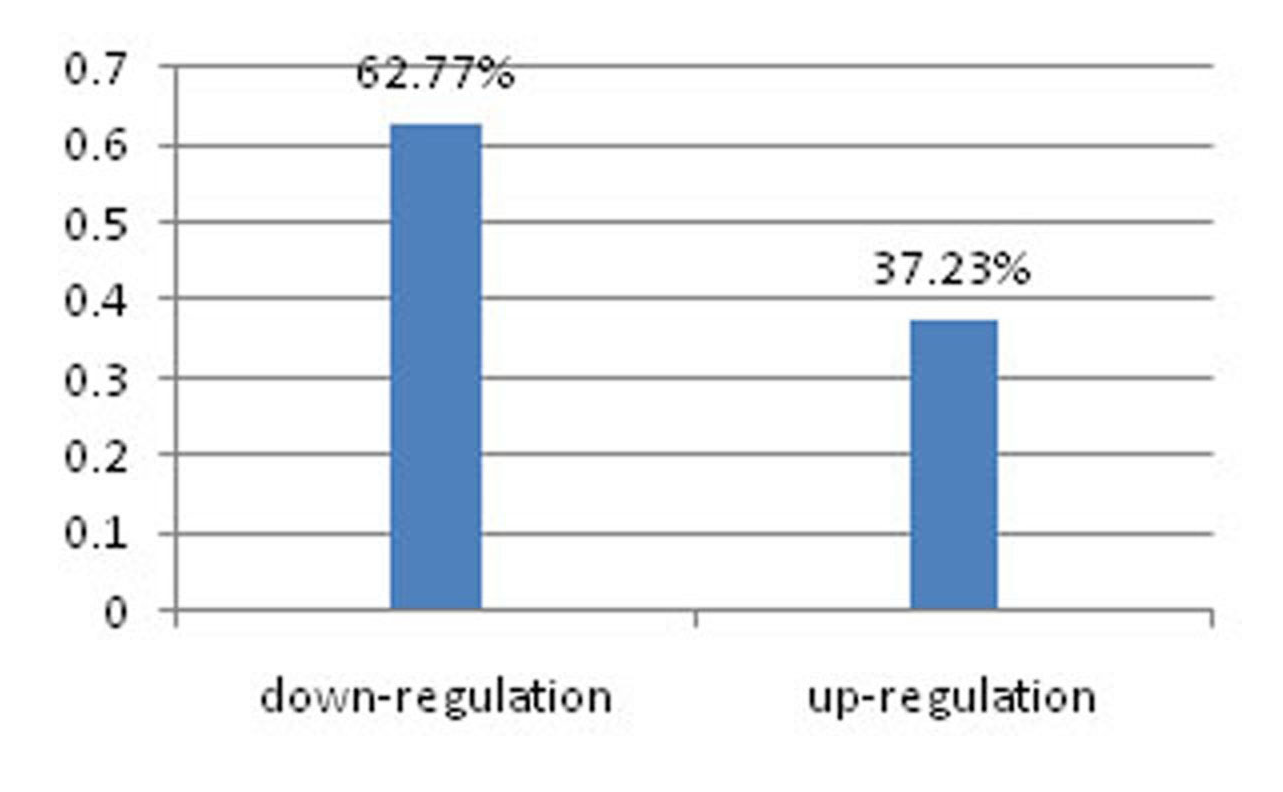
**Percentage of down- and up- regulatory interactions identified by SA-BNs**. In the 231 statistically significant interactions, 145 are down-regulatory interactions, and 86 are up-regulatory interactions.

Several miRNAs have recently been described as crucial regulators of EMT and metastasis. Apart from the up-regulatory mechanism of miRNAs, down-regulations have also been identified in several works. For example, Gebeshuber et al. [20] found up-regulation of miRA-29a in mesenchymal, metastatic RasXT cells relative to epithelial EpRas cell. Liu et al. [19] found that miRNA-146a was up-regulated in human bronchial epithelial cells. The results from SA-BNs suggest that more up-regulation of miRNAs could be in EMT. In the 231 statistically significant interactions, there are 145 interactions are down-regulation, while 86 interactions are up-regulation (Figure 6).

**Figure 7:**
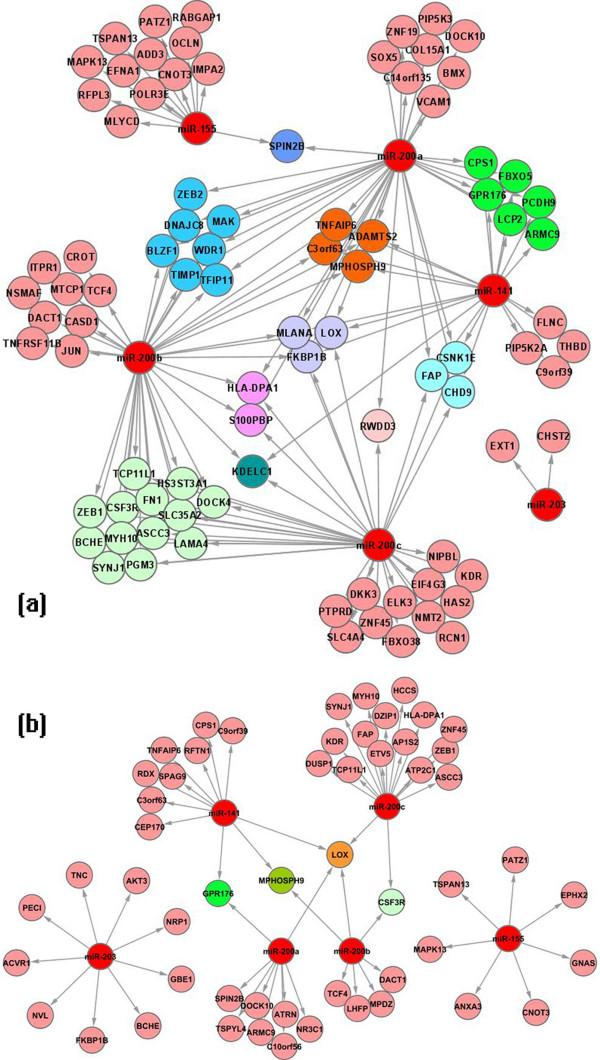
**Discovered miRNA-mRNA interaction networks**. miRNA-mRNA interaction networks discovered by (a) SA-BNs and (b) normal BNs for EMT, rendered in Cytoscape [49]. In these modules, the miRNA-mRNA pairs are negatively correlated. miRNA are colored by red. The mRNAs co-targeted by same miRNAs are grouped and shown in the same color.

We first focus on negatively correlated miRNA-mRNA pairs. Figure 7-(a) is the significant miRNA-mRNA interaction network for EMT with only down-regulated interactions.

#### Validating targets with TarBase and miRecords

We validate the results of SA-BNs against two experimentally supported databases, TarBase V5.0 [44] and miRecords [45]. As shown in Table 2, the number of experimentally validated targets for the identified miRNAs is very small. In total, 16 target relationships consisting of 6 miRNAs and 12 mRNAs have experimental supports from TarBase and miRecords. Among them, 5 target relationships are supported by our experimental data sets and also predicated by SA-BNs.

**Table 2:** Validating targets against TarBase and miRecords

miRNA	Target gene	Predicated by SA-BNs for EMT	Validated Interaction for EMT	Support Database	Pubmed ID
miR-200a	*ZEB2/SIP1*	*	*	TarBase, miRecords	18376396

miR-200a	*ZEB1/TCF8*		*	TarBase, miRecords	18376396

miR-200b	*RERE*			miRecords	17923093

miR-200b	*ZEB1/TCF8*	*	*	TarBase, miRecords	18376396

miR-200b	*ZEB2/SIP1*	*	*	miRecords	18376396

miR-200c	*ZEB1/TCF8*	*	*	TarBase, miRecords	18483486

miR-200c	*ZEB2/SIP1*	*	*	TarBase	18381893

miR-141	*CLOCK*			TarBase, miRecords	15131085

miR-141	*TGF-β*		*	miRecords	18483486

miR-155	*AGTR1/AT1R*			TarBase, miRecords	16675453, 17668390, 7588946

miR-155	*BACH-1*			TarBase, miRecords	17881434

miR-155	*LDOC1*			TarBase, miRecords	17881434

miR-155	*MATR3*			TarBase, miRecords	17881434

miR-155	*TM6SF1*			TarBase, miRecords	17881434

miR-203	*SOCS-3*			miRecords	17622355

miR-203	*P63*			miRecords	18483491

16 target relationships consisting of 6 miRNAs and 12 mRNAs have experimental supports from TarBase and miRecords. Among them, 5 out of 7 identified miRNA-mRNA interactions by SA-BNs have been confirmed experimentally for EMT (denoted by *).

It is worth noting that SA-BNs is mainly designed to indentify the miRNA-mRNA interactions for specific conditions. In the analysis, it has been used to discover the miRNA-mRNA interactions for EMT. Table 2 shows that 5 out of 7 identified miRNA-mRNA interactions by SA-BNs have been confirmed experimentally for EMT. It suggests that SA-BNs are promising to discover the miRNA-mRNA interactions for specific conditions. In the following, we will discuss the interactions for EMT in detail.

#### SA-BNs discover the miR-200 family target ZEB1 and ZEB2 for EMT which have been experimentally validated

The miR-200 family has been identified to play a central role in the regulation of the epithelial to mesenchymal transition [28,29,46]. In the interaction network, SA-BNs identified experimentally validated targets of *miR-200 *family. The results of SA-BNs show that *ZEB1 *is co-targeted by *miR-200b *and *miR-200c*, and *ZEB2 *is co-targeted by *miR-200a *and *miR-200b *in EMT module at statistically significant level. Correlation tests show that miR-200 negatively correlates with *ZEB1 *and *ZEB2 *at significant level (*p*-value < 0.005, adjusted by BH method). The discovery indicates that the miR-200 family negatively regulates *ZEB1 *and *ZEB2*, in agreement with previous experimental work showing that the miR-200 family regulate EMT by directly targeting *ZEB1 *and *ZEB2 *[28,29,46]. This discovery of SA-BNs is consistent with the validated results.

#### SA-BNs discover LOX has wide interactions with miR-200 family for EMT which is also supported by literature

SA-BNs show that *LOX *is negatively co-regulated by all *miR-200 *family members inducing EMT. Higgins et al. have suggested that *LOX *regulates EMT [47]. This is consistent with our results and suggests that *LOX *has wide interactions with the miR-200 family members for EMT.

#### A significant number of mRNAs identified by SA-BNs participate in the biological processes of EMT

The functions of identified mRNAs and the molecular pathways they potentially constitute were assessed by using Ingenuity Pathway Analysis (IPA) software. The regulated targets are significantly enriched for several biological functions. The top five functions listed by IPA are known to be critical for EMT. They are cellular development, cell-to-cell signaling and interaction, cellular movement, gene expression, and cellular growth and proliferation. Specifically, sub-categories of cellular movement, migration, invasion, and scattering, have been identified as the functional markers of EMT [42]. In the results identified by SA-BNs, a significant number of mRNAs associate with these EMT functional markers (Table 3), details in Additional file 3). It suggests that SA-BNs captured many mRNAs and their interactive miRNAs participating in EMT.

**Table 3:** Identified mRNAs are significantly involved in the functional markers of EMT

Functions	Molecules	Number	*p*-value
migration	*ADAM12, ADRB2, BMX, CSF3R*,	17	1.56 × 10^-4 ^- 1.65 × 10^-2^
	*CTBP2, EFNA1, FAP, FN1*,		
	*HAS2, IL6, KDR, LOX, MYH10*,		
	*PTPRU*, *TIMP1, VCAM1, ZEB2*		

invasion	*DKK3, FN1, HAS2, JUN, LOX*,	9	3.74 × 10^-3 ^- 1.15 × 10^-2^
	*TIMP1, YY1, ZEB2, MYH10*		

scattering	*EFNA1, FN1*	2	4.98 × 10^-3^

In the results identified by SA-BNs, a significant number of mRNAs associate with these EMT functional makers.

#### Molecular networks participated in by identified mRNAs are highly relevant to EMT, suggesting that the pathways of identified mRNAs may also be regulated by the miR-200 family

**Figure 8:**
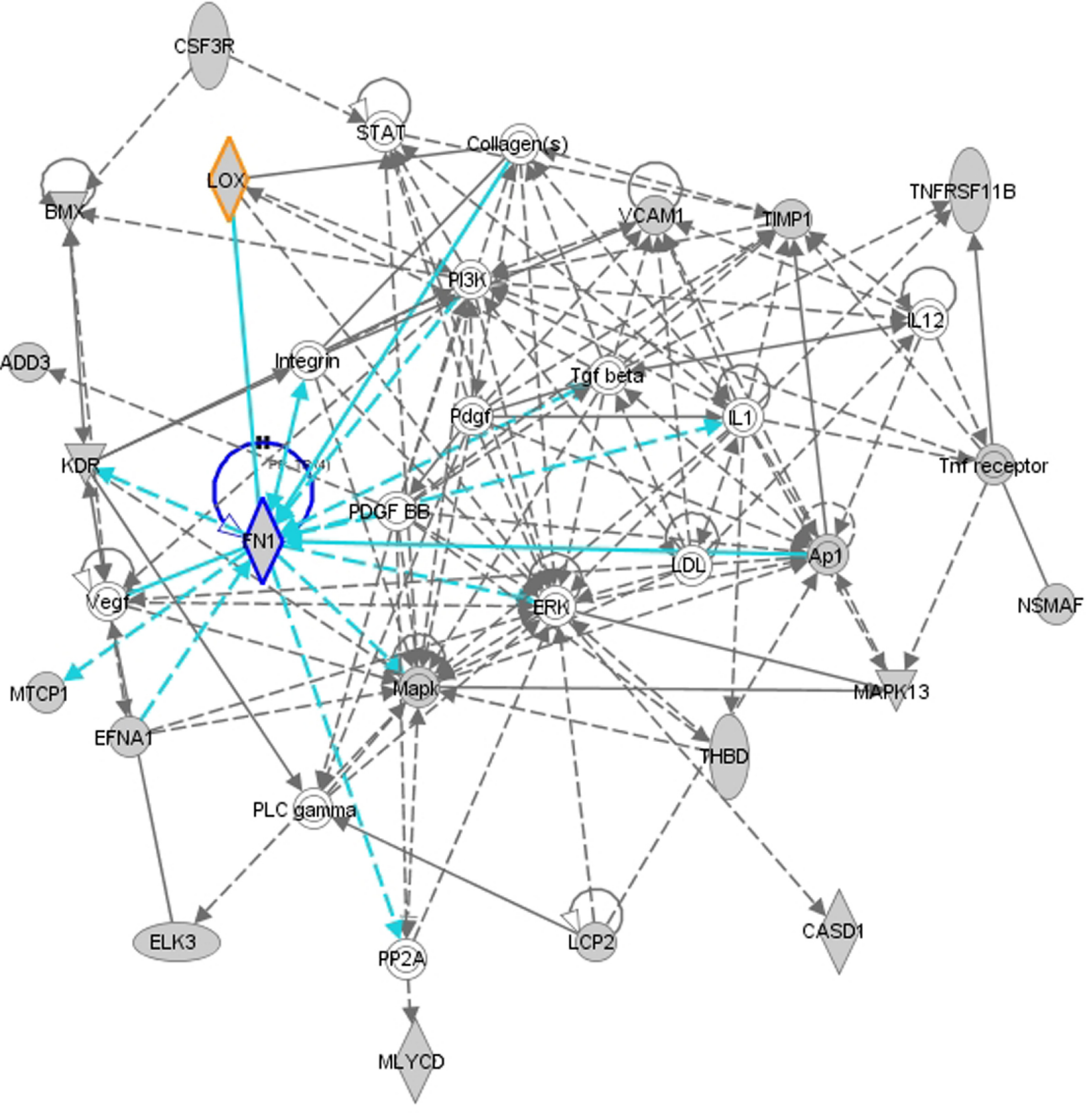
**A molecular network with functions in cancer, cellular movement, and gastrointestinal disease**. 18 out of 34 mRNAs (colored in grey) in this network are identified by SA-BNs. Highlighted in graph, *FN1 *is a hub interacting with many other molecules involved in functions associated with EMT. SA-BNs suggest that miR-200b and miR-200c co-regulate *FN1 *for EMT. In addition, *LOX*, which was identified to interact with miR-200 family by SA-BNs, also involves in this network by interacting with many other molecules, including *FN1*.

**Figure 9:**
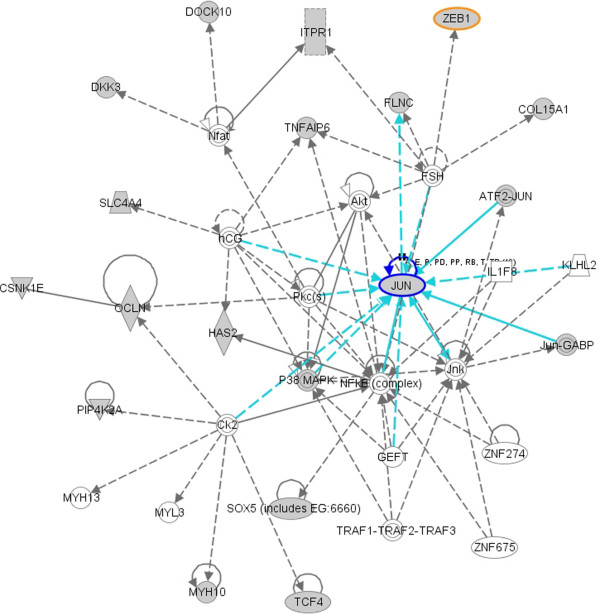
**A molecular network with function in cell morphology, skeletal and muscular system development and function, connective tissue development and function**. 16 out of 35 mRNAs (colored in grey) in this network are identified by SA-BNs, including one of the validated targets of miR-200 family, *ZEB1 *(highlighted). In addition, *JUN*, regulated by *miR-200b *according to SA-BNs, interacts with many other molecules involved in apoptosis, proliferation, growth, transformation, cell death, morphology, cell cycle progression, survival, colony formation, and motility.

**Figure 10:**
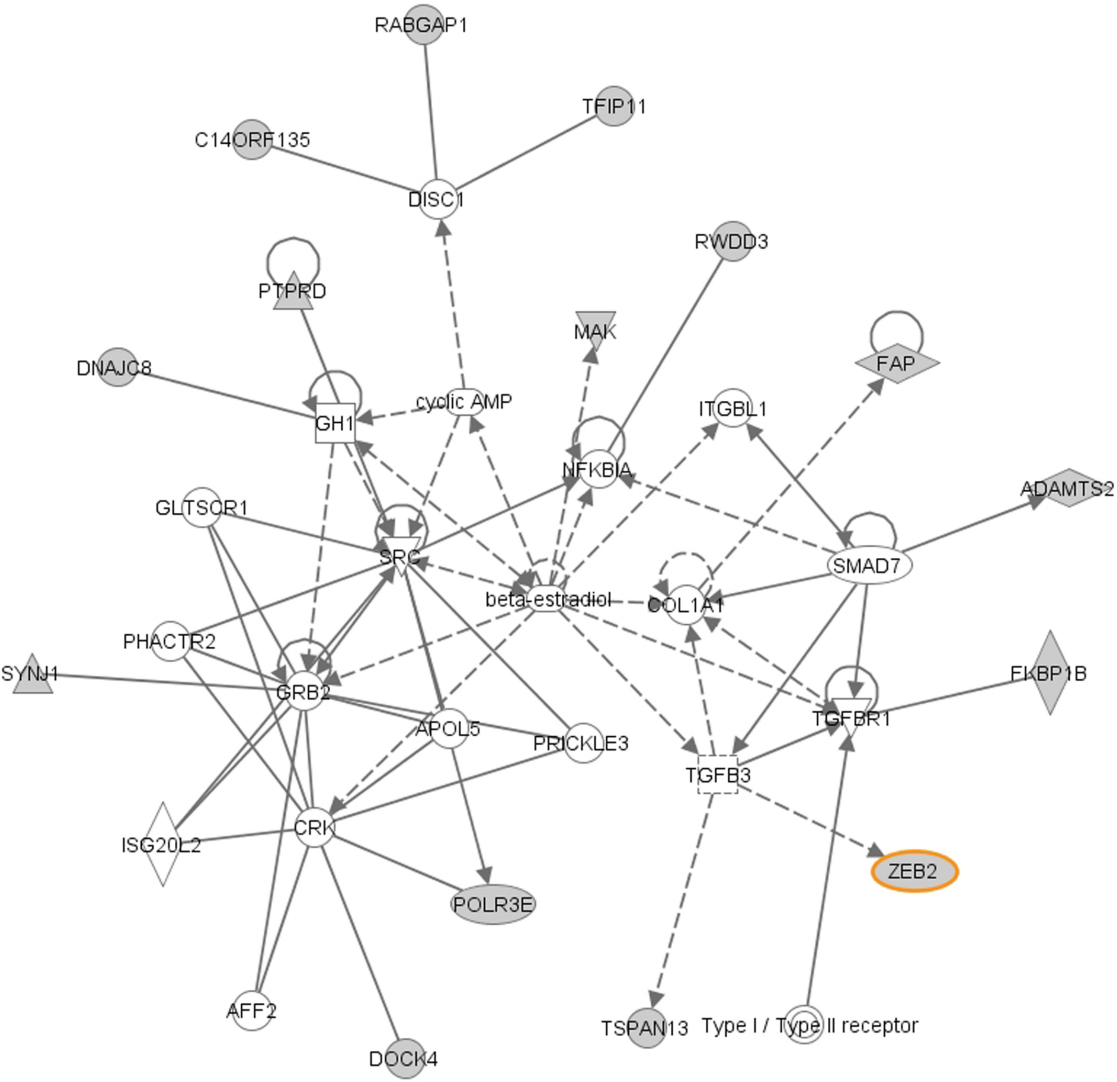
**A molecular network with function in cellular growth and proliferation, cellular function and maintenance respiratory system development and function**. 15 out of 35 mRNAs (colored in grey) in this network are identified by SA-BNs, including one of the validated target of miR-200 family, *ZEB2 *(highlighted).

IPA identified 8 molecular networks constituted by the 88 predicted targets which are down-regulated by miRNAs, exemplified by three networks which are highly relevant to EMT in Figure 8, 9 and 10. The network in Figure 8 suggests functions in cancer, cellular movement and gastrointestinal disease. 18 out of 34 mRNAs in this network are identified by SA-BNs. In the network, *FN1 *is a hub interacting with many other molecules involved in functions associated with EMT, including migration, adhesion, cell spreading, apoptosis, proliferation, formation, attachment, quantity, assembly, and invasion. SA-BNs suggest that miR-200b and miR-200c co-regulate *FN1 *for EMT. In addition, *LOX*, which was identified to interact with miR-200 family by SA-BNs, is involved in this network by interacting with many other molecules, including *FN1*. Figure 9 shows the network has functions in cell morphology, skeletal and muscular system development, and connective tissue development. 16 out of 35 molecules in this network are identified by SA-BNs, including one of the validated targets of miR-200 family, *ZEB*1. Furthermore, *JUN*, regulated by *miR-200b *according to SA-BNs, interacts with many other molecules involved in apoptosis, proliferation, growth, transformation, cell death, morphology, cell cycle progression, survival, colony formation, and motility. Figure 10 is the network with functions in cellular growth and proliferation, cellular function and maintenance. 15 out of 35 of mRNAs in this network are identified by SA-BNs, including one of the validated targets of miR-200 family, *ZEB2*. Mechanisms mediated by these molecules have been implicated in EMT. Figure 8, 9 and 10 suggest that these pathways may also be regulated by the miR-200 family.

### Comparing Networks Identified by SA-BNs and Normal BNs

We compared the miRNA-mRNA interactions discovered by SA-BNs to those identified by normal Bayesian networks under the same settings. With normal BNs, 98 miRNA-mRNA interactions were identified at statistically significant level. They comprise 6 miRNAs and 84 mRNAs (Additional file 4). The significant interaction network with only negatively correlated modules is given in Figure 7-(b). In this network, normal BNs identified only one validated miR-200 target, *ZEB1*.

#### The topology of interaction network identified by SA-BNs is more biologically appropriate than that of normal BNs

In comparison with the network identified by SA-BNs (Figure 7-(a)), the network identified by traditional BNs is more sparse. SA-BNs capture more mRNAs that are potentially co-targeted by multiple miRNAs, which is a biological expectation when the miRNAs are known to contribute to the same biological process, as is the case for the multiple members of the miR-200 family [29]. Furthermore, based on their sequence similarity in the "seed region", miR-200a and miR-141 are predicted to interact with the same target sites. miR-200b and miR-200c, which share identical 5' ends, are predicted to recognize another set of targets in common [29]. However, in the network discovered by normal BNs, only one mRNA is co-targeted by miR-200a and miR-141, and only 2 by miR-200b and miR-200c. In contrast, 16 mRNAs are co-regulated by miR-200a and miR-141, and 19 mRNAs are co-regulated by miR-200b and miR-200c in the network discovered by SA-BNs. Thus the network from SA-BNs gives a more expected result than the one from normal BNs.

#### SA-BNs discover more relevant miRNA-mRNA interactions for EMT

**Figure 11:**
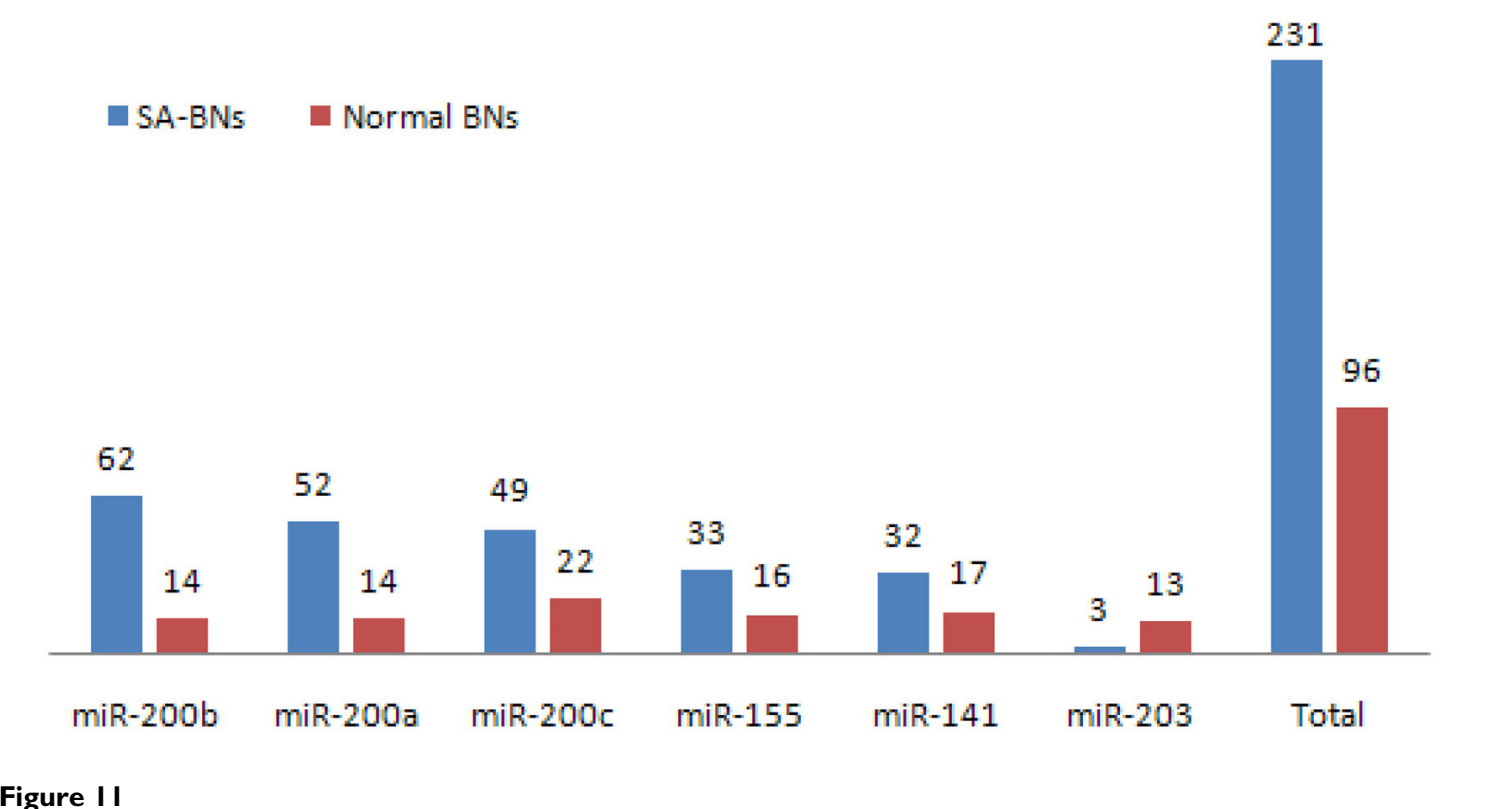
**Number of miRNA-mRNA interactions discovered by SA-BNs and a normal Bayesian network method**. The number of statistically significant interactions for each miRNA and the total number of interactions discovered by SA-BNs and normal BNs. SA-BNs discovered more statistically significant interactions than a normal BN.

Figure 11 shows the number of interactions for each miRNA and the total number of interactions discovered by SA-BNs and normal BNs. SA-BNs discovered more statistically significant miRNA-mRNA interactions than normal BNs.

To determine whether the unique set of interactions discovered by SA-BNs has different patterns which are specific to SA-BNs, we reviewed the correlations of miRNA-mRNA samples for each category, that is, epithelial and mesenchymal. It shows that a large number of miRNA-mRNA pairs show inconsistent correlation patterns across sample categories. For example, SA-BNs captured that miR-200c interacts with *FN1 *while the normal BNs did not. At the data level, miR-200c and *FN1 *show positive correlation in epithelial samples, but negative correlation in mesenchymal samples. The inconsistent patterns of local correlations prevent the normal BNs from discovering subtle interactions between miRNAs and mRNAs. SA-BNs are able to discover both strong and subtle interactions while the data show inconsistent patterns through available samples.

To determine whether the unique set of mRNAs discovered by SA-BNs is biologically relevant to EMT, we inquired IPA the different sets of mRNAs discovered by SA-BNs and normal BNs. It shows that the number of mRNA uniquely discovered by SA-BNs is more than that of the normal BNs in terms of EMT relevant functions, including the EMT functional markers (Table 4). It suggests that SA-BNs capture more EMT relevant miRNA-mRNA interactions compared with normal BNs.

**Table 4:** Comparison of results between SA-BNs and normal BNs

EMT relevant cellular functions	SA-BNs		Normal BNs	
	
	#Molecules	*p*-value	#Molecules	*p*-value
Cellular Movement	14	4.62 × 10^-4 ^- 2.86 × 10^-2^	7	9.83 × 10^-4 ^- 3.92 × 10^-2^
Cell Morphology	17	2.49 × 10^-4 ^- 2.86 × 10^-2^	10	1.74 × 10^-4 ^- 4.47 × 10^-2^
migration*	6	4.62 × 10^-4 ^- 2.86 × 10^-2^	6	6.93 × 10^-3 ^- 3.59 × 10^-2^
invasion*	6	1.53 × 10^-3 ^- 2.46 × 10^-2^	1	3.76 × 10^-2^
scattering*	2	1.27 × 10^-3^	1	1.74 × 10^-3^

The number of mRNA uniquely discovered by SA-BNs is more than that of the normal BNs in terms of EMT relevant functions, including the EMT functional markers denoted by *.

## Discussion

In the past few years, the identification of miRNAs and their targets has made significant progress. Current focus is shifting to the elucidation of miRNA functions. However, some specific features of miRNAs, for example their small size, abundance of repetitive copies and mode of action, pose several challenges in studying of miRNA functions [48].

miRNAs show diverse regulatory mechanisms with mRNAs. They have been known to down-regulate target mRNAs in the majority of cases. The up-regulation of miRNA also has been reported recently [17,18], and even down- and up-regulations depending on physiological conditions [17]. The various observations of miRNA regulation make it difficult to generalize simple rules for miRNA-mRNA interactions, especially under different physiological conditions. Most previous work has studied the discovery of down-regulatory modules of miRNAs and mRNAs by computational methods [22,25]. The up-regulatory and mix-regulatory mechanisms of miRNAs have not been identified from existing data. However, the discovery of up- and mix-regulatory mechanisms reveal the complex interactions of miRNAs and mRNAs, such as indirect regulations. Considering that most biological experiments have been designed for a comparative study, such as normal versus malignant, down- and up-regulatory mechanisms, especially featuring in the different phenotypes, conditions, or treatment groups, are of great interest to medical researchers.

In this work, we propose a new Bayesian network structure learning method to explore all types of miRNA-mRNA interactions by using heterogeneous information. Much research has been done to discover the gene regulatory networks with BNs on homogeneous data, for instance, microarray data or protein data, but not much work has been done to discover the interactions between miRNAs and mRNAs. Apart from making use of heterogeneous information such as miRNA-target binding, expression profiles of miRNAs and mRNAs, and sample categories, an innovation of the proposed method is to design a splitting and averaging scheme for Bayesian structure learning to discover up- and down-regulatory mechanisms of miRNAs. In addition, small sample size is a problem for reliable discoveries. We use bootstrapping and a statistical model to obtain reliable probability estimation of interactions discovered by SA-BNs.

Bootstrapping alleviates the overfitting problem which is common for machine learning on small size of data sets. The false discovery is well controlled by bootstrapping and the constraint of miRNA-target prediction.

The proposed method finds many regulatory mechanisms that have been supported by previous research. For example, the discovery of the miR-200 family targeting *ZEB1 *and *ZEB2 *for EMT has experimentally validated in previous research [28,29,46]. Other discoveries are also very promising. For instance, the results of SA-BNs show *LOX *widely interacts with the miR-200 family for EMT. It is consistent with previous research which suggests *LOX *regulates EMT [47]. In addition, the significant number of identified mRNAs have biological functions in EMT, especially the marker functions of EMT like migration, invasion, and scattering. It suggests that SA-BNs have captured many mRNAs and their interactive miRNAs participating in EMT. Furthermore, many molecular networks participated in by identified mRNAs are highly relevant to EMT, suggesting that the pathways of identified mRNAs may also be regulated by the miR-200 family.

The regulatory networks from our method reveal more mRNAs co-regulated by multiple miRNAs than a normal Bayesian network does. Multiple interactions are consistent with the current view of complex regulatory mechanism of miRNAs. Though there is no direct evidence to support the discovered up-regulatory and mix- regulatory mechanisms for EMT from previous research, this work indicates that there are many of such interactions supported by data at statistically significant levels. One reason is that little research has been conducted on this new area yet. These differentially regulatory mechanisms among different conditions are of great interest. We expect they can be validated by biological experiments in the near future.

## Conclusions

In this study, we have proposed a method to explore the complex miRNA-mRNA interactions with Bayesian networks by a splitting-averaging strategy. It is designed to discover both strong and subtle interactions from expression profiles of miRNAs and mRNAs under the constraints of a putative targeting database. Several issues of BNs have been addressed, including integration of heterogeneous data, constraints of the BNs structures with prior knowledge, overfitting, and model integration with splitting and averaging. The analysis of EMT data sets shows that SA-BNs discover more biologically relevant miRNA-mRNA interactions compared to normal BNs. Some discoveries have been validated by previous research. Some are consistent with the literature. Some are statistically significant interactions that are novel and worthy of validation by biological experiments in the near future.

## Authors' contributions

BL and JYL conceived of this research. BL designed and performed the experiments. AT, AG, and GG provided the miRNA data and validated the results. LL verified the mathematical model. BL and JYL drafted the manuscript. All authors read and approved the final manuscript.

## Supplementary Material

Additional file 1 **Differentially expressed miRNAs and mRNAs**. Welch *t*-statistics with 10,000 times permutation is used to identified differentially expressed miRNAs and mRNAs. The selected miRNAs and mRNAs are highlighted. Probe names, miRNA names, test statistic, *p*-values and Benjamini and Hochberg adjusted *p*-values are listed. Click here for file

Additional file 2 **Significant miRNA-mRNA interactions identified by SA-BNs**. SA-BNs identify 231 statistically significant interactions which comprise 127 unique mRNAs and 6 miRNAs for EMT. miRNA-mRNA pairs, confidence, Pearson's correlation coefficients of miRNA-mRNA pairs within and across sample categories are listed. Click here for file

Additional file 3 **The biological functions participated in by identified mRNAs**. In the results identified by SA-BNs, a significant number of mRNAs associate with these EMT functional makers (highlighted in the table). It suggests that SA-BNs captured many mRNAs and their interactive miRNAs participating in EMT. Click here for file

Additional file 4 **Significant miRNA-mRNA interactions identified by normal BNs**. Normal BNs identify 98 statistically significant interactions which comprise 84 unique mRNAs and 6 miRNAs for EMT. miRNA-mRNA pairs, confidence, Pearson's correlation coefficients of miRNA-mRNA pairs within and across sample categories are listed. Click here for file
